# A comparison study between GeXP-based multiplex-PCR and serology assay for Mycoplasma pneumoniae detection in children with community acquired pneumonia

**DOI:** 10.1186/s12879-017-2614-3

**Published:** 2017-07-25

**Authors:** Le Wang, Zhishan Feng, Mengchuan Zhao, Shuo Yang, Xiaotong Yan, Weiwei Guo, Zhongren Shi, Guixia Li

**Affiliations:** 1 Institute of Pediatric Research, Children’s Hospital of Hebei Province, 133 Zhonghua South Street, Shijiazhuang, Hebei Province 050031 China; 2 Department of Laboratory Medicine, Children’s Hospital of Hebei Province, Shijiazhuang, 050031 China

**Keywords:** *Mycoplasma pneumoniae*, Community acquired pneumonia, Multiplex-PCR, Serology

## Abstract

**Background:**

Diagnosis of community-acquired pneumonia (CAP) caused by *Mycoplasma pneumoniae* (Mp) in children has been hampered by difficulty in obtaining convalescent serum and time constraints. In this study, the two diagnostic assays that targeted respectively on Mp-antibody and Mp-DNA were retrospectively investigated.

**Methods:**

A total of 3146 children were clinically diagnosed to have CAP and were confirmed by chest X-ray during March 2015 to February 2016 in Children’s hospital of Hebei Province (China). Both of the sera and sputum samples were collected in 24 h after their admission. The Mp-antibody was examined by the passive particle agglutination assay and a fourfold or greater increase of antibody titers of paired sera or≧1:160 titer of single serum was set as the serology positive. Mp-DNA in the sputum samples was tested by a multiplex-PCR method named GeXP assay (multiplex PCR combined with automated capillary electrophoresis). In order to eliminate the false positive results caused by the asymptomatic carriage after infected by *M. pneumoniae,* the inconsistent samples were tested by the real-time isothermal transcription-mediated RNA amplification assay (SAT).

**Results:**

The inter-rated agreement test was performed in 3146 CAP patients, with a highest kappa value in the school-age children as 0.783. There were 6.29% (198/3146) cases showed inconsistent results determined by GeXP and serology assay. All of the 19 GeXP(+)/Serology (−) samples and a randomly chosen 27 from 179 GeXP(−)/Serology (+) samples were tested by SAT assay, and a 97.8% diagnosis agreement was observed between SAT and GeXP assay, but not with the serology assay. In addition, patients who were detected only by serology or only by multiplex-PCR were significantly younger than those with both methods positive (3.0 and 1.5 years vs. 5.0 years, *p* < 0.01). The Viral-Mp coinfection accounted for 37.0% (97/262), which was more common in winter and spring (*p* < 0.05) and in the infantile group (*p* < 0.01), compared to the pure Mp positive ones.

**Conclusion:**

In some children CAP cases, the Mp laboratory diagnosis was inconsistent between serology and multiplex-PCR assay. Verified by the SAT assay, the GeXP showed a more sensitive and reliable performance compared with the serology assay. Furthermore, employing the multiplex-PCR could provide more information on the associated pathogens for clinical assessment of CAP.

**Electronic supplementary material:**

The online version of this article (doi:10.1186/s12879-017-2614-3) contains supplementary material, which is available to authorized users.

## Background

*Mycoplasma pneumoniae* (Mp) is a leading cause (about 40%) of community-acquired pneumonia (CAP) especially in the pediatric populations [1]. The golden diagnostic criteria of *M. pneumoniae* infection has been considered as the seroconversion or rising IgG titers [2], but the antibody results are susceptible to the children’s age and immunity status [3, 4], especially facing to the difficulty in obtaining convalescent serum in a pediatric hospital [5].

Due to the advantages of PCR on its sensitivity, specificity and early detection, large body of literature has been published for using PCR to detect Mp-DNA or RNA, including conventional, nested, real-time, multiplex and isothermal amplification PCR [6]. Among these, the multiplex-PCR has been accepted as a more practical diagnosis method in *M. pneumoniae* CAP, because the existence of clinical similarity resulted from Mp and other agents [7] and the common appearance of the viral co-infections [8]. Thus, in the recent study, the *M. pneumoniae* was added into our previously used pathogen panel that included 20 types/subtypes of viruses [9]. Meanwhile, several other groups have also designed the multiplex-PCR for the early detection of *M. pneumoniae* infection [10–17], but so far, the direct comparison between multiplex-PCR and serology test in larger pediatric clinical samples has rarely been reported [6]. Besides, the *M. pneumoniae* RNA detection (real-time isothermal transcription-mediated RNA amplification assay, Mp-SAT) that targeted on the specific 16SrRNA has been invented and investigated [18, 19], which could eliminate the false positive cases resulted from the previous infection or the nonpathogenic carrier state [20].

In this study, sputum samples were obtained and analyzed by a commercial multiplex-PCR kit named GeXP multiplex amplification assay for 13 types/subtypes pathogens (including *M. pneumoniae*) that were commonly detected in patients with CAP. The passive particle agglutination (PA) assay was used to examine the same patients’ Mp-antibody in their serum specimens. A comparison between these two assays was conducted, and the Mp-SAT assay was used to test the inconsistent samples. The possible factors that may contribute to the disagreement results were also analyzed.

## Methods

### Study population

The protocols used in this retrospective study was reviewed and approved by the institutional review board of Children’s Hospital of Hebei Province. The written informed consent was obtained from each patient’s parent prior to enrollment. We selected specimens over a 1-year period (2015–2016) to cover all four seasons. Based on CAP Diagnosis and Management Guidelines [21], a total of 3146 otherwise healthy children aged 0.1–16.0 years (median, 2.0 years) were confirmed by the chest X-ray and finally enrolled. They respectively received the Mp-antibody and Mp-DNA testing within 24 h of admission. Patients with congenital heart or lung disease, or with immunosuppression, or had received the immunosuppressive therapy, or had been admitted to a hospital longer than 2 days within the last 90 days were excluded.

### Detection of mp-antibody by the passive particle agglutination assay

The paired serum samples were taken at the presentation of pneumonia and at least 7 days after the first collection of serum. The serum was obtained from 2 mL whole blood by the separation gel tube. And the determination of Mp-specific antibody was performed using a commercially available micro-particle agglutination test Serodia-MycoII kit (Fujirebio, Tokyo, Japan) according to the manufacturer’s instructions [22]. Antibody titers were measured at a range of dilutions (from 1:40 to 1:20,480). Criteria for diagnosis were defined as > or =4-found rising for paired sera of *M. pneumoniae* antibody [2] or single serum of titer > or =1:160 [23, 24].

### Sputum specimen collection

Patients were asked to cough and the expectorated sputum was collected. If the child is too young to cough, a sterile negative pressure suction catheter is applied to obtain the oropharyngeal suction (OPS) into transport tube containing 1 ml DMEM medium with 2% heat-inactivated fetal calf serum, 50 IU/ml of penicillin and 100 μg/ml of streptomycin (Gibco, Beijing, China). The sample was stored at 4 °C for the same day pathogen nucleic acid extraction. Total nucleic acid (both DNA and RNA) was extracted from 200 μL sputum sample and eluted into 30 μL nuclease-free water by the EasyPure Viral DNA/RNA Kit (QSJBio, Beijing, China) in accordance with the manufacturer’s instructions. Afterwards, 3 μL of extracted nucleic acid was analyzed with the GeXP-based assay. The remaining sputum samples were added into equal volume of preservative solution and stored at a − 80 °C freezer.

### Detection of mp-DNA by the automated GenomeLab GeXP genetic analysis system

The GeXP assay (GenomeLab GeXP Genetic Analysis System) was performed on all specimens for the following 13 different respiratory pathogens,: Influenza A (Flu A), Influenza B (Flu B), Influenza A H1N1 pdm09 (09H1), influenza H3N2 (H3), human parainfluenza virus (HPIV), respiratory syncytial virus (RSV), rhinovirus (HRV), adenovirus (ADV), human metapneumovirus (HMPV), human bocavirus (HBoV), human coronavirus (HCoV), *Chlamydia* (*Ch*) and *Mycoplasma pneumoniae* (*Mp*), using the 13 Respiratory Pathogens Multiplex Kit (PCR-Capillary Electrophoresis Fragment Analysis) (Health Gene Tech., Ningbo, China). The reverse transcription (RT) and PCR were performed as previously described in Wang, et al. [9]. The analysis was then performed in an automated manner following the established protocol and the data were compiled by the GeXP system software provided by Beckman Coulter.

### Detection of mp-rRNA by SAT

The frozen sputum specimens were thawed on the day the assays were performed. RNA extraction was performed on the MagX automated platform (Rendu Biotech., Shanghai, China), in which the targeted 16SrRNA could be enriched and purified by the magnetic particles. The final RNA was eluted into 30 μL nuclease-free water. The negative and positive controls were also introduced into the next Mp-SAT procedure. The reaction was performed as follows: incubation at 60 °C for 10 min and 42 °C for 5 min, 10 μL of SAT enzyme was added into the reaction. The Mp-SAT was completed on a real-time thermocycler (Veriti Thermal Cycler, Applied Biosystems China) in following steps: step1, 42 °C for 1 min; step2 was repeated for 40 cycles. The fluorescent light channel was set as FAM (sample channel) and HEX (internal control channel). The amplified products were then assessed using the SAT analysis system (Rendu Biotech), and a real-time PCR Ct value≦30 is defined as positive.

### Statistical analysis

The Pearson and Linear-by-linear association chi-squared test was used to compare sex, admission season and age subgroups. The Kruskal-Wallis test was used to compare mean age at onset of illness. SPSS 13.0.1 statistics package (SPSS Inc., Chicago, USA) software was used for all statistical analysis. *p* < 0.05 was considered statistically significant.

## Results

### *M. pneumoniae* detection by serology versus multiplex-PCR testing

Sputum and serum specimens collected from 3146 hospitalized children were tested by GeXP assay and PA assay, respectively. A total of 243 samples (7.72%, 243/3146) were both positive and 2705 samples (85.98%, 2705/3146) were both negative based on these two methods. The inconsistent samples accounted for 6.29% (198/3146). The Kappa value was ordinary (κ = 0.677, *p*< 0.01), and the highest kappa value (κ = 0.783) was observed in the school-age children group (Table 1).

**Table 1:** The consistency of test results for *M. Pneumoniae* between serology and multiplex-PCR testing

	MX-PRC	Serology		Kappa	*P*
		+	-	Value	
0-3 yr	+	52	14	0.486	<0.01
	-	78	855		
3-6 yr	+	81	3	0.723	<0.01
	-	51	910		
>6 yr	+	110	2	0.783	<0.01
	-	50	942		
Total	+	243	19	0.677	<0.01
	-	179	2705		

To better understand the discordant samples, the terms of ‘false positive’ and ‘false negative’ rates of serology were introduced. The premise to use them is to assume the results of multiplex-PCR as gold standard. If the sample is tested to be Serology (+)/Multiplex-PCR (−), it is presented as ‘false positive’; to be Serology (−)/Multiplex-PCR (+), it is presented as ‘false negative’. We found that both of the ‘false positive’ and ‘false negative’ rates of serology were significantly (*p*< 0.001) associated with age (Table 2). The ‘false positive’ samples took a proportion of 60% (78/130) in ages 0–3, 39% (51/132) in 3–6 years, and 31% (50/160) in ages >6 years. Similarly, ‘false negative’ samples accounted for 21% (14/66), 4% (3/84) and 2% (2/112) in ages 0–3, 3–6 and >6 years old, respectively.

**Table 2:** The age-dependent false positive and false negative rates of serology assay

Age	False positive rate	Statistic value	*P*	False negative rate	Statistic	*P*
					value	
0–3 year	60% (78/130)			21% (14/66)		
3–6 year	39% (31/132)	23.532^a^	<0.001	4% (3/84)	20.500^a^	<0.001
>6 year	31% (50/160)			2% (2/112)		

^a^ Linear-by-Linear Association

Of the total 422 patients with positive serology results, paired sera were collected from 26 patients (6.2%). Interestingly, the 26 paired sera samples were in good agreement with multiplex-PCR results: 92% (24/26) cases with four fold increase titer which were corresponded to the multiplex-PCR positive.

In addition, children in whom *M. pneumoniae* was detected by serological or by multiplex-PCR alone were significantly younger than those who were detected by both methods (Z = −4.20, *p*< 0.01). But no significant differences were observed for their admission season (*p* = 0.165) or sex (*p* = 0.338) (Table 3).

**Table 3:** Characteristics of *M. Pneumoniae* detection by serology and multiplex-PCR testing

Characteristics		Values of the Cases Tested				
		Serology (+) /MX-PCR(+)	Serology (−) /MX-PCR(+)	Serology (+) /MX-PCR (−)	Statistic value	*P*
		(*n* = 243)	(*n* = 19)	(*n* = 179)		
Age (Median)		5.0	1.5	3.0	35.423^a^	<0.01
Season (n)	Spring	27	2	35	9.150^b^	0.165
	Summer	42	2	28		
	Autumn	53	7	33		
	Winter	121	8	83		
Sex (n)	Male	142	12	93	2.169^b^	0.338
	Female	101	7	86		

^a^ Kruskal-Wallis test

^b^ Pearson Chi-Square

### Testing of specimens with mp-SAT assay

A total of 46 cases with inconsistent results were re-analyzed by the Mp-SAT assay. We randomly chose 27 samples from 179 cases that had been determined to be Serology(+)/MX-PCR (−), together with the whole 19 Serology (−)/MX-PCR(+) cases. Among the 46 sputum samples redo the Mp-SAT assay, we found 97.8% diagnosis agreement between SAT and GeXP assay. A total of 18 Serology (−)/MX-PCR (+) cases were determined to be SAT positive, and the whole 27 Serology(+)/MX-PCR (−) cases were all tested to be SAT negative (Additional file 1 Table S1).

### Mp coinfection with other pathogens

Ninety-seven patients (37.0%, 97/262) had other causative pathogens besides *M. pneumoniae*. Among these patients, HRV was the most detected (20.2%, 53/262), followed with HPIV, FluB, ADV, RSV, 09H1, H3, HCoV, HBoV and HMPV (Table 4).

**Table 4:** The rates of co-infection of *M. pneumoniae* CAP patients by other respiratory pathogens

	Pathogens	Positive Num.
	No other pathogens	165 (63.0%)
1	HRV	53 (20.2%)
2	HPIV	15 (5.7%)
3	FluB	12 (4.6%)
4	ADV	9 (3.4%)
5	RSV	9 (3.4%)
6	09H1	8 (3.1%)
7	H3	5 (1.9%)
8	HCoV	4 (1.5%)
9	HBoV	4 (1.5%)
10	HMPV	2 (0.8%)
11	*Ch*	0 (0.0%)

In the comparison of two patients groups that infected only with *M. pneumoniae* and mixed infected with other viruses, the significant discrepancies existed in different season and age subgroups, but not in the sex groups (Table 5).

**Table 5:** Comparison of age, season and sex characteristics between children with only M. pneumoniae and those with mixed infection by at least one respiratory virus

			Positive rate (n)		Statistic value	*P*
		N	Mp only	Mp w/t virus		
Sex	Male	154	61.7% (95)	38.3% (59)	0.27^a^	0.606
	Female	108	64.8% (70)	35.2% (38)		
Season	Spring	32	68.8% (22)	31.2% (10)	5.12^b^	0.023
	Summer	43	72.1% (31)	27.9% (12)		
	Autumn	61	72.1% (44)	27.9% (17)		
	Winter	126	54.0% (68)	46.0% (58)		
Age	0–1 yr	16	25.0% (4)	75.0% (12)	18.96^b^	<0.01
	1–3 yr	50	46.0% (23)	54.0% (27)		
	3–6 yr	83	67.5% (56)	32.5% (27)		
	>6 yr	113	72.6% (82)	27.4% (31)		

^a^ Pearson Chi-Square

^b^ Linear-by-Linear Association

The seasonal distribution analysis revealed that, patients infected only with *M. pneumoniae* were more than the mixed infection patients during summer and autumn. But in spring and winter, the mixed infection patients showed to be predominate (χ^2^ = 5.12, *p* = 0.023). Interestingly, the positive detection rate of school-age children was highest in the *M. pneumoniae* only group, but the lowest in the mixed infection group (χ^2^ = 18.96, *p*< 0.01).

## Discussion

The nucleic acid amplification techniques (NAATs) targeted towards DNA or RNA have increasingly been explored for identification of pathogens including *M. pneumoniae* in infectious respiratory diseases [10–17]. So far, only a few studies have described the application of NAATs for pathogen detection in children with CAP [25–27]. Determination as well as comparison between laboratory diagnosis methods of *M. pneumoniae* infection for childhood CAP in larger clinical database has not been reported. In the present study, we applied a multiplex-PCR, named GeXP assay, to detected 13 types of pathogens including *M. pneumoniae* in 3146 sputum samples from hospitalized CAP children. Meanwhile, their serum specimens were also collected to be tested the antibody against *M. pneumoniae.* After the comparison between serology and GeXP assays on the same patient, about 93.7% of cases were diagnosed to be either positive or negative based on these 2 methods. We randomly chose 46 samples from 198 samples that had been tested to be inconsistent by serology and GeXP assays, redo the Mp-SAT and found 97.8% diagnosis agreement between SAT and GeXP assays, but not with the serology testing. Further analysis revealed that children in whom *M. pneumoniae* was only examined to be positive by GeXP or serology were significantly younger than those of both methods positive. About 37% of patients have virus-Mp coinfection, which were most observed in infantile patients. Seasonal distribution analysis revealed that the virus-Mp coinfection were predominant in spring and winter, while Mp-only infection was more frequently identified in summer and autumn.

Only a few reports have thus far described the comparison between serology and mono-PCR assays for *M. pneumoniae* detection in pediatric CAP patients [28–31]. Using the paired sera samples, three independent research groups all observed excellent diagnosis agreement between these 2 methods [28–30], in contrast to our findings and Kim’s [31]. This discrepancy is mainly attributed to the proportion of paired sera. Because in our results, only 13% patients provided their second sera specimen, the good concordance could be observed between these paired sera with multiplex-PCR, On the other hand, after comparing the mono-PCR and serology assay, Kim proposed that a single titer ≧1:640 were considered to indicate the acute *M. pneumoniae* infection [31]. But in clinical, a presumptive diagnosis could be made from a single acute-phase serum titer ≧1:160 [24]. Therefore, if we interpret the Multiplex-PCR positive samples as truly indicating Mp infection, the high ‘false positive rate’ of serology testing (42%, 179/422) may caused by the proportion of paired sera and the criteria of diagnosis. Besides that, the serology ‘false positive rate’ was probably due to the other two aspects: Firstly, the serology assay is claimed to detect both of Mp-IgM and IgG [22], the positive serology results may be caused by an earlier (not current) Mp infection, which is still detectable in the blood. Alternatively, the current infection truly exists, but the amount of organism was below the detection limit of multiplex-PCR. It has been reported that the diagnostic accuracy of PCR may decrease at ≥7 days after onset of disease in contrast to serology [32], especially patients who receive week duration of the antibiotic treatment [31].

Besides these mono-PCR studies, as reviewed by Loens et al. [6], several groups have applied the multiplex-PCR in the detection of *M. pneumoniae* infection, but the comparison assays were set as culture and/or mono-PCR, which are more suitable for accuracy validation during the methodology establishment process rather than obtaining the clinical diagnostic significance. In addition, Due to the high viral coinfection prevalence reported by Chen (25.9% [33]) and ours (37.0%), it is essential to recommend the use of multiplex-PCR assay to provide more pathogen information. More importantly, in Mp IgM-positive children, a negative PCR result was reported to be associated with coinfection by other pathogens [32]. Our results also demonstrated that the youngest children group exhibited highest Serology(+)/Multiplex-PCR (−) rate and highest coinfection rate. In young children, since the serology positive cases may be result from other pathogen infection, the multiplex-PCR should be used to rectify these sorts of false positive cases determined by serology. Furthermore, a number of studies have demonstrated a weak or deferred antibody response to *M. pneumoniae* in young children [31–36], suggesting a possible reason leading to the highest Serology (−)/Multiplex-PCR (+) rate observed in the youngest patients group in our study. In conclusion, owing to the high coinfection rate and weak antibody response in young children, the Mp infection is needed to be monitored not only by the serology assay. A combination of serology and multiplex-PCR allows for both decreased ‘false positive’ and ‘false negative’ rates of serology assay.

In the present study, the inconsistent results were observed and took a 6.29% proportion, beside of 90% Multiplex-PCR (−)/Serology (+) samples mentioned above, the remaining 10% discordant samples were Multiplex-PCR (+)/Serology (−). Previous work has shown that the colonization of *M. pneumoniae* may cause the false positive results [37–39]. To rule out the possible false positive results of multiplex-PCR (targeting Mp-DNA) caused by colonization, the SAT assay (targeting Mp-RNA) were applied and we found that a overwhelming majority (94.7%, 18/19) of Multiplex-PCR (+)/Serology (−) sputum specimens were proved to be SAT positive. On the contrast, the randomly selected Multiplex-PCR (−)/Serology (+) sputum specimens were all observed to be SAT negative. These data suggest that the multiplex-PCR assay would be more reliable when the serology assay exhibits the opposite results. However, we felt a verification experiment with larger sample size should be used in future work. Another limitation of this work is that the highly specialized equipment is needed, namely an automated nucleic acid extraction system, which could carried out 48 samples at one time in about 1 h. Many primary hospitals or clinical laboratories do not have this equipment, and the manual work would greatly increase the turnaround time.

## Additional file

Additional file 1: Table S1. The Ct value of 18 Serology (−)/MX-PCR (+) cases. (DOC 35 kb)

## Abbreviations

09H1: Influenza A H1N1 pdm09
ADV: Adenovirus
CAP: Community acquired pneumonia
*Ch*: *Chlamydia*
FluA: Influenza virus A
FluB: Influenza virus B
GeXP assay: GenomeLab Genetic Analysis System
H3: Influenza H3N2
HBoV: Human bocavirus
HCoV: Human coronavirus
hMPV: Human metapneumovirus
HRV: Human rhinovirus
*Mp or M*: *Pneumoniae*, *Mycoplasma pneumoniae*
MX-PCR: Multiplex-PCR
NAATs: Nucleic acid amplification techniques
PA assay: Particle agglutination assay
PIV: Parainfluenza virus
RSV: Respiratory syncytial virus
RT-PCR: Reverse transcription polymerase chain reaction
SAT: Real-time isothermal transcription-mediated RNA amplification assay
