# Supplementary material for: A comparison study between GeXP-based multiplex-PCR and serology assay for Mycoplasma pneumoniae detection in children with community acquired pneumonia
Source: BMC Infect Dis. 2017 Jul 25;17:518. doi: 10.1186/s12879-017-2614-3 (PMC5527399; doi:10.1186/s12879-017-2614-3)
Supplement: Additional file 1: Table S1. — The Ct value of 18 Serology (−)/MX-PCR (+) cases. (DOC 35 kb) [file 12879_2017_2614_MOESM1_ESM.doc]

Table S1. The Ct value of 18 Serology(-)/MX-PCR(+) cases.

| Serology(-)/  MX-PCR(+) | Ct value |
| --- | --- |
| 1 | 11.1 |
| 2 | 10.1 |
| 3 | 10.5 |
| 4 | 14.4 |
| 5 | 10.5 |
| 6 | 12.7 |
| 7 | 10.4 |
| 8 | 9.6 |
| 9 | 11.8 |
| 10 | 14.1 |
| 11 | 11 |
| 12 | 10.9 |
| 13 | 11.2 |
| 14 | 10.6 |
| 15 | 14.8 |
| 16 | 10.1 |
| 17 | 12.2 |
| 18 | 9.5 |
